# Examining indicators of complex network vulnerability across diverse attack scenarios

**DOI:** 10.1038/s41598-023-45218-9

**Published:** 2023-10-24

**Authors:** Ahmad F. Al Musawi, Satyaki Roy, Preetam Ghosh

**Affiliations:** 1 Department of Information Technology, University of Thi Qar, Thi Qar, Iraq; 2 Department of Computer Science, Virginia Commonwealth University, Richmond, VA USA; 3 Department of Mathematical Sciences, The University of Alabama in Huntsville, Huntsville, AL USA

**Keywords:** Computer science, Computational science

## Abstract

Complex networks capture the structure, dynamics, and relationships among entities in real-world networked systems, encompassing domains like communications, society, chemistry, biology, ecology, politics, etc. Analysis of complex networks lends insight into the critical nodes, key pathways, and potential points of failure that may impact the connectivity and operational integrity of the underlying system. In this work, we investigate the topological properties or *indicators*, such as shortest path length, modularity, efficiency, graph density, diameter, assortativity, and clustering coefficient, that determine the vulnerability to (or robustness against) diverse attack scenarios. Specifically, we examine how node- and link-based network growth or depletion based on specific attack criteria affect their robustness gauged in terms of the largest connected component (LCC) size and diameter. We employ partial least squares discriminant analysis to quantify the individual contribution of the indicators on LCC preservation while accounting for the collinearity stemming from the possible correlation between indicators. Our analysis of 14 complex network datasets and 5 attack models invariably reveals high modularity and disassortativity to be prime indicators of vulnerability, corroborating prior works that report disassortative modular networks to be particularly susceptible to targeted attacks. We conclude with a discussion as well as an illustrative example of the application of this work in fending off strategic attacks on critical infrastructures through models that adaptively and distributively achieve network robustness.

## Introduction

Complex network theory is a field of study that investigates connectivity patterns in large networks and explores the interactions among entities^1^. These networks, such as social networks, technological connections, and biological networks, offer valuable insights into the structure and dynamics of diverse systems. Understanding the structures of complex networks holds significance for multiple reasons. It enables researchers and scientists to analyze and model the behavior of real-world systems. By examining connectivity patterns and indicators, we can gain insights into the underlying mechanisms that govern these systems. Complex network analysis aids in the identification of pivotal nodes, termed *hubs* or influencers, that assume critical roles in network dynamics and information flow within the network. By understanding their importance, decision-makers can devise better strategies for optimizing connectivity, services, and functionalities^2^.

Studying complex networks helps detect *vulnerabilities* and potential points of failure in diverse and large-scale networked systems^3^. It is essential to identify weak links or nodes that, if disrupted, can impair network functionality, enabling the design of robust systems. This knowledge finds application in domains like power grids, transportation systems, and communication networks. Complex network analysis also reveals insights into information dissemination, disease spread, and behavioral patterns within networks. Understanding how information or influences propagate aids in developing strategies to improve diffusion processes and control epidemics^4^. Furthermore, complex network structures guide the design of efficient and scalable networks. By examining complex network connectivity and growth criteria, researchers can optimize performance, resource allocation, and routing strategies of large-scale networked systems through algorithm and protocol development^5^.

There are innumerable examples of network vulnerability in the real world. These include the distribution of viruses in communication networks, the rapid spread of epidemics in societies, unexpected failures of servers or routers, disruptions in power links, road cuts in transportation networks, and disruptions in fuel distribution networks. The networking community has explored the design of *robust* networks that employ redundancy, fault tolerance, and adaptive mechanisms to overcome their vulnerability to different attack scenarios^6^. For instance, the analysis of network performance after deactivating a set of nodes and/or edges falls under the regime of percolation theory on networks. Several researchers studied network percolation in terms of fragility (vulnerability) and robustness of the network against random or predefined attacks^7–11^. Salathe et al.^12^ analyzed the scale-free^13^ connectivity property of networks through a model that uses selective node removal based on the inverted sum of the first and second order of connectivity (i.e., number of neighbors of 1 and 2 hubs distances) followed by random addition of nodes to the network. Holme et al.^14^ has studied the performance of different networks under attack (in terms of the size of the largest connected subgraphs) using two node removal criteria: descending order of node’s degree and node’s betweenness. Their study showed that the attacks on the updated degree and betweenness centralities of nodes are more harmful than those of the initial networks. Iyer^15^ extended targeted nodes to more non-local measures of importance such as degree distribution, clustering coefficient, and assortativity. Smith et al.^16^ address the problem of finding the optimal order of repairing elements in power grids and similar infrastructure after catastrophic events. The paper concludes that high structural redundancy and decentralized supply in infrastructure systems can lead to reduced total cost and faster recovery time.

Several generative models are proposed to mimic the structure of given real networks, such as Erdos-Renyi model^17^, the small world model^18^, preferential attachment^13^, the Barabasi and Albert model^13^ and so on. Safaei et al.^19^ proposed a rewiring mechanism based on the Shannon entropy concept to improve the resiliency of complex networks. Network robustness was evaluated based on the spectrum of the degree distribution, heterogeneity, and the average size of the largest connected cluster during removing nodes with a sequence of systematic attacks based on the degree, betweenness, and Dangalchev’s closeness centralities. With approximately 30% of link rewiring, overall network robustness can be reached. Other works have mixed two or more mechanisms to depict real-world features, such as the mixing of clustering and preferential attachment^20^, popularity and randomness^21^, popularity and similarity^22^ or topological and geographical measures^23^ and so on. More research extended the study of network robustness to consider larger structures such as motifs^24^ and subgraphs^25^. For these studies to be effective, it is imperative to quantify the contribution of network properties to the overall robustness.

**Figure 1:**
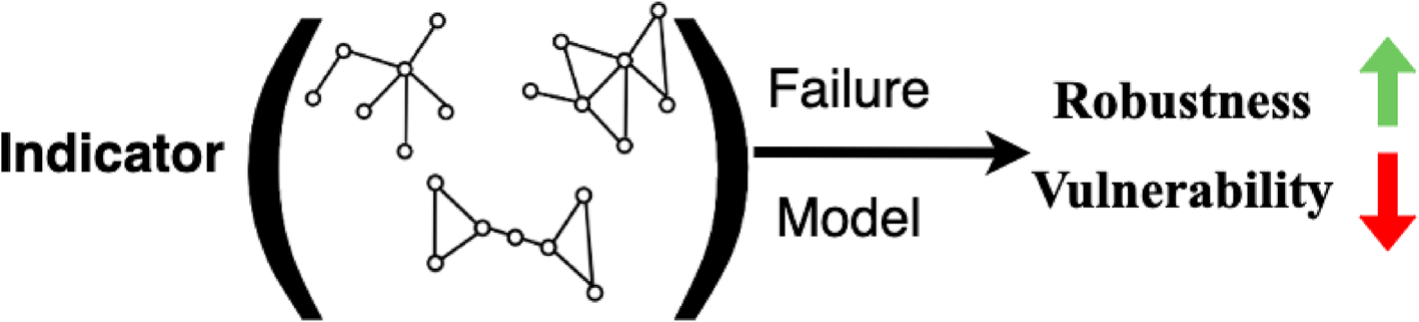
A schematic representation of the approach adopted in this study, where the goal is to pinpoint the topological properties or *indicators* that explain the robustness or vulnerability of complex networks to diverse network attack models.

In this paper, we study the topological properties or *indicators* of complex networks that determine their vulnerability to (or robustness against) different attack scenarios. We borrow the concept of *robustness* from literature as the ability of a network to preserve connectivity despite the removal of components, i.e., nodes and links; and the absence of this ability as *vulnerability*^26^. We first study how the robustness of complex networks is affected by the choice of attack strategy such as connectivity, betweenness, closeness, clustering, etc., of the attacked components. We then study whether the presence or absence of some topological indicators (namely, shortest path length, modularity, efficiency, graph density, diameter, assortativity, and clustering coefficient) may explain why networks exhibit resilience against the chosen attack strategy. Fig. 1 depicts a schematic of the above approach, where we analyze the indicators rendering networks robust or vulnerable to attacks.

We examine network vulnerability in three ways. *First*, we consider a depletion model, where the links in each network are sequentially depleted based on attack strategies of interest while recording the change in the largest connected component (LCC) size with respect to the original networks. We deem a network vulnerable to a specific attack scenario if it fragments quickly into smaller components. Similarly, we also carry out the reverse experiment of recording how quickly a network attains its maximum size of LCC when it is grown from an empty graph by sequentially restoring all links based on their scores for a given attack criteria. For the growth model experiments, a robust growing network is likely to attain its maximum LCC size faster than a vulnerable one. *Second*, we employ the vulnerability of a network from a node-removal standpoint in terms of the change in network diameter as well as node- and link-based robustness when nodes are knocked off the network. *Third*, we employ *partial least squares discriminant analysis* (PLS-DA) to quantify the contribution of topological indicators as exogenous variables on the preservation of LCC during link depletion attacks on networks. Since PLS-DA combines discriminant analysis with principal component analysis, it accounts for the *collinearity* problem caused by the possible correlation between independent variables and helps pinpoint the individual role of an indicator on overall network robustness (gauged through LCC).

We apply 7 topological indicators, namely, average shortest path, assortativity, density, diameter, clustering coefficient, efficiency, and modularity (refer to section Topological indicators in complex networks) and 5 attack models, which are random, degree, betweenness, closeness, and clustering (refer to section Network attack models) on 14 complex network datasets (see section Results). Network modularity and assortativity emerge as key indicators in all three vulnerability analyses, followed by clustering and density. Note that PLS-DA has been conducted on quantile-transformed values of each topological indicator, making the contributions of the indicators on network robustness relative. In other words, *a high coefficient of assortativity for a given attack scenario does not necessarily indicate that assortative networks are more robust for that attack. Instead, it can mean that the lack of disassortativity is a useful indicator of robustness, i.e., a network that is neither assortative nor disassortative exhibits greater robustness than a disassortative network.* Lastly, we discuss how this line of study motivates the design of approaches to preserve robustness under specific attack scenarios. We focus on its application towards guarding against attacks on critical infrastructures, such as smart grids, telecommunication networks, etc., and PLS-DA as a viable tool to pinpoint threats to network integrity (see sections Application of the study and Discussion).

## Methods

### Topological indicators in complex networks

The assessment of a network’s ability to withstand various types of attacks can differ depending on its topological characteristics. Specifically, a network is deemed robust if it retains a strong level of connectivity, despite failures. Numerous network features are available to characterize the type of network under consideration. The following set of features are well-known topological-based network features/properties that are used to distinguish the differences among networks^27^:

1. *Averaged shortest path* (ASP) is the average number of hops along the shortest paths for all possible pairs of network nodes. It is calculated as:
  1 $$\begin{aligned} ASP = \sum \limits _{s,t \in V} \frac{d(s,t)}{n(n-1)} \end{aligned}$$
  Here *V* is the set of nodes, *d*(*s*, *t*) is the shortest path from *s* to *t* and *n* is the number of nodes.
2. *Assortativity*^28,29^ measures the tendency of nodes to have a connection with other nodes that are similar in degree (among many other features). In other words, assortativity ranges between $0 \le r \le 1$ when high-degree nodes are most likely to connect to high-degree nodes while it is the same for low-degree nodes. Also, we get ($-1 \le r \le 0$), if high-degree nodes make connections to low-degree nodes. It is calculated as:
  2 $$\begin{aligned} r = \frac{\sum _{ij}(A_{ij}-k_i k_j/2m)k_i k_j}{\sum _{ij}(k_i \delta _{ij}-k_i k_j/2m)k_i k_j} \end{aligned}$$
  Here, *A* is the adjacency matrix of the network, $k_i$ is the degree of node *i*, and $\delta _{ij}$ is the Kronecker delta.
3. *Density* measures the number of edges in comparison to network size. A network has a zero density if it has no edge, and it has a density of 1 if there is an edge between all pairs of nodes, forming a complete graph. It is calculated as:
  3 $$\begin{aligned} d = \frac{2m}{n(n-1)} \end{aligned}$$
  where, *m*, *n* is the number of edges and nodes, respectively.
4. *Diameter* represents the maximum shortest path distance among all pairs of nodes. Since the diameter of a graph with many components is not defined, in this study we measure the diameter of the largest connected component.
5. *Transitivity* also known as the global clustering coefficient or transitive closure, is a measure that quantifies the tendency of nodes in a network to form triangles or closed loops. It provides an indication of how likely it is for two neighbors of a node to be connected to each other, given that the node itself is already connected to them.
  4 $$\begin{aligned} T = 3 \times \frac{\# triangles}{\#triads} \end{aligned}$$
  Here, the triad represents two edges with a shared node.
6. *Efficiency*^30^: The efficiency of a pair of nodes in a graph refers to the reciprocal of the shortest path distance between those nodes. It quantifies how easily information can flow between the nodes. The average global efficiency of a graph is determined by calculating the average efficiency across all possible pairs of nodes in the graph. It measures the overall effectiveness of information transfer in the graph, taking into account the efficiency of all connections between nodes, as:
  5 $$\begin{aligned} e = \frac{1}{n(n-1)} \times \sum _{i,j \in V} \frac{1}{d(i, j)} \end{aligned}$$
  where, *n* is the number of nodes, *d*(*i*, *j*) denotes the shortest path distance between node *i* and node *j*, $\sum _{i,j \in V} \frac{1}{d(i, j)}$ signifies the summation of the reciprocal of the shortest path distances for all pairs of nodes *i* and *j* in the graph.
7. *Modularity*^31^: It is a measure that quantifies the degree of community structure or clustering in a graph. It assesses the extent to which nodes in a network are more connected to nodes within their own community compared to nodes in other communities. Higher modularity values indicate a stronger community structure, with nodes being tightly connected within their communities and sparsely connected across different communities. It is calculated as follows:
  6 $$\begin{aligned} M = \frac{1}{2m} \times \sum (A_{ij} - \frac{k_i \times k_j}{2m}) * \delta (c_i, c_j) \end{aligned}$$
  where, $A_{ij}$ denotes the adjacency matrix element, representing the connection between nodes *i* and *j*. $k_i$ and $k_j$ represent the degrees of nodes *i* and *j*, respectively. *m* represents the total number of edges in the network. $\delta (c_i, c_j)$ is the Kronecker delta function, equal to 1 if nodes *i* and *j* belong to the same community ($c_i = c_j$), and 0 otherwise.
8. *Node robustness* ($R_n$) and *edge robustness* ($R_e$) measure the connectivity of the network when subjected to the removal of nodes and links, respectively^32,33^.
  7 $$\begin{aligned} R_n = \frac{1}{|V|} \sum _{q = \frac{1}{|V|}} ^ {1} S(q) \end{aligned}$$
  8 $$\begin{aligned} R_e = \frac{1}{|E|} \sum _{p =\frac{1}{|E|}} ^{1} S(p) \end{aligned}$$
  Here, |*V*| is the number of nodes, *S*(*q*) is the fraction of nodes in the largest connected subgraph after the removal of *q*|*V*| nodes (or *p*|*E*| edges), and *q* is the fraction of nodes to be targeted from the remaining nodes in |*V*| (or edges |*E*|).

### Network attack models

Networks can be vulnerable to different types of attacks where network components like nodes or edges are removed based on predefined schemes. These attacks can happen randomly or deliberately, like virus attacks, and may depend on specific factors. To model network attacks, various scenarios are proposed, falling into three basic categories: deleting nodes, deleting edges, and deleting groups of nodes and edges known as motifs. In the following explanation, we focus on node deletion attacks, but the same principles apply to edge removals as well.

1. *Random attack* (RND): removes a specific percentage of nodes randomly.
2. *Degree-based node attack* (DNA) creates a list of nodes for removal based on the descending order of the nodes’ degree.
3. *Betweenness-based node attack* (BNA): creates a list of target nodes for removal based on the descending order of the nodes’ betweenness centrality^34^. The betweenness of node (*v*) is given by Eq. (9).
  9 $$\begin{aligned} Betweenness(v) = \sum _{s\ne v\ne t \in V} \frac{\sigma _{st}(v)}{\sigma _{st}} \end{aligned}$$
  Here, (*s*, *t*) are pair of nodes, $\sigma _{st}$ is the shortest path between *s*, *t* and $\sigma _{st}(v)$ is the fraction of shortest paths that pass through node *v*.
4. *Closeness-based node attack* (CNA) creates a list of targets based on the descending order of the nodes’ closeness centrality. (Closeness Centrality^35^, as shown in Eq. (10), measures the average proximity of a node with respect to all other nodes. High closeness centrality-scored nodes have the shortest distance to all other nodes.)
  10 $$\begin{aligned} Closeness(v) = \frac{1}{\sum _{i \ne v}d_{vi}} \end{aligned}$$
  Here, $d_{vi}$ represents the distance from node *v* to node *i*.
5. *Clustering-based node attack* (CcNA) creates a list of node targets based on the descending order of the nodes’ clustering coefficient values. The clustering coefficient^36^ measures the local clustering of nodes, i.e., the connection tendency between two unconnected nodes sharing a connection to a common node. The clustering coefficient of node (*v*) is:
  11 $$\begin{aligned} ACC = \frac{1}{|V|}\sum _v {C_v} \end{aligned}$$
  Here, $C_v$ is the ratio between the number of triplets connected to node *v* and the number of triplets centered on *v*.

These methods of attack have varying impacts on network connectivity. Degree-based node attack (DNA) works on removing the most influential nodes that have the highest connections within the network. DNA attack reduces the number of edges very fast. Betweenness-based node attack (BNA) affects the communication or connectivity of the networks. It works on dividing the network into unconnected subgraphs as its target nodes exist on the shortest paths between all pairs of nodes. Closeness-based node attack (CNA) works on removing nodes that have the highest access to most nodes of the network.

### Network growth and depletion models

Growth and depletion models are used to analyze network vulnerability. In a growth model, we begin with a disconnected set of nodes and restore links based on prespecified criteria, while observing the improvement in overall connectivity, measured by the size of the largest connected component. Conversely, for network depletion, we follow the reverse process of starting with the network itself and dropping links till all nodes are isolated. The criteria for growth or depletion are as follows:

#### Centrality-based ranking of edges

An edge $e_{x,\,y}$ is considered for addition to the network based on its high specific-degree weight. A degree weight of an edge is equal to the product of the specific degree of its two nodes, (*x*, *y*). We used four centralities to grow the network: degree centrality, closeness, betweenness, and clustering coefficient centrality of nodes. For each, the edge weights are calculated as:

12 $$\begin{aligned} w_{x,y}^{m} = Centrality_m (x) \times Centrality_m (y) \end{aligned}$$

Here, *x*, *y* are nodes and *m* refers to one of the centralities in use (degree, betweenness, closeness, and clustering coefficient). All resulting edges are sorted in descending order. The network grows by adding the highest weighted edges (of associated nodes) to the network.

#### Centrality-based preferential attachment model

We extend the three variations to the well-known model of preferential attachment^13^, considering betweenness, closeness, and clustering coefficient centralities. In the preferential attachment model, a node is most likely to form a connection (link) based on the proportional value of its degree to the total degrees of the network:

13 $$\begin{aligned} \Pi (k_i) = \frac{k_i}{\sum _j k_j} \end{aligned}$$

Here, $k_i$ represents the degree of node *i*, resulting in the “rich become richer” phenomenon where highly connected nodes received more connection than other nodes. As before, we use closeness, betweenness, and clustering coefficient values as a method of linking instead of using the node’s degree. For example, two (unconnected) nodes of high betweenness centrality would have a higher probability to link/connect than nodes with lower betweenness values, $\Pi (B_i) = \frac{B_i}{\sum _j B_j}$ where $B_i$ represents the betweenness of node *i*. The new centrality-based preferential attachment models will be the betweenness-based preferential attachment (BPA) model, closeness-based preferential attachment (CPA) model, and clustering-based preferential attachment (CcPA) model. We also performed another criterion to grow the network given the degree of the nodes. However, we used an inverted version of the PA model, i.e., low-weighted edges are added first to the network based on the following equation:

14 $$\begin{aligned} iPA_{i,j} = \frac{1}{k_i k_j}, i,j \in V, i\ne j. \end{aligned}$$

The growth (and depletion) models are employed to test how the robustness of the network evolves as links are restored (and eliminated). There are two key points in the growth strategy followed in this study. (Note that the same steps are emulated during the link removal phase in the depletion model.)

1. Estimating the changing centrality attributes after the addition of every link is computationally expensive. To address this, we split the edges into 100 groups. Instead of recalculating centrality for every added link, we use a pre-sorted list of edges, ranked by centrality, and add a batch of edges based on the original network’s centrality values.
2. The addition of edges in this study is not influenced by the previous state of the network (i.e. centralities). Instead, we solely focus on the weighted edges of the original network to expand the network in a similar manner. This approach allows us to examine the network’s robustness as it progressively restores its initial structure by prioritizing the addition of influential edges. It is worth noting that there are alternative methods for network growth that take into account the current centrality of the network during the process of redistributing edges. However, we chose not to incorporate these growing methods as they would result in the creation of different network versions.

**Figure 2:**
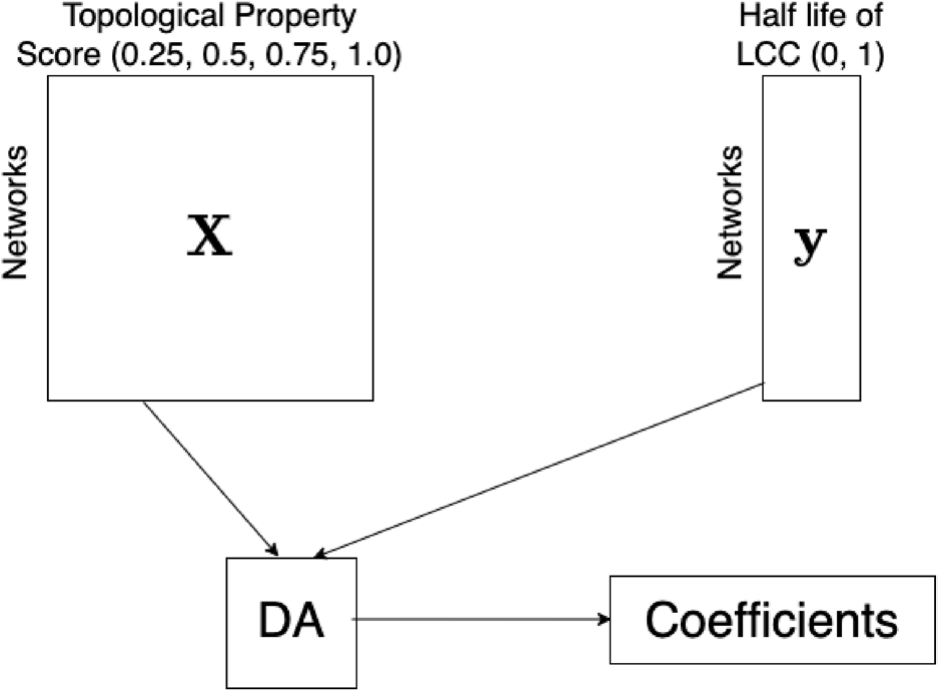
Partial least squares discriminant analysis (DA) coefficients calculated from the features consisting of all network indicators and labels gauging the number of link removals to make the largest connected component half its original size.

### Partial least squares discriminant analysis

The partial least squares (PLS) regression technique employs dimensionality reduction that works by projecting the independent variables onto a latent space. PLS regression is particularly useful for finding the effect of independent variables on a dependent variable in scenarios where the independent variables are mutually correlated (also called multicollinear predictors). PLS discriminant analysis is a special case of PLS regression, where the dependent variable is categorical.

We analyze the effect of the topological indicators of a network on its vulnerability to different failure scenarios. To this end, we start with each network and remove edges in batches in sequences given by predefined preferential attachment (PA) criteria (refer to section Network growth and depletion models), namely, degree (DPA), betweenness (BPA), closeness (CPA), clustering coefficient (CcPA), random (RND) and inverted preferential attachment (iPA). The network growth stops when all links are eliminated from the network. For every PA criterion, we carry a standalone discriminant analysis with the following features and labels.

#### Features

The features comprise the topological indicators: transitivity, modularity, density, assortativity, average shortest path, and diameter. A column in the feature vector $\textbf{X}$ is dedicated to one of the topological indicators across *n* networks. We discretize each feature value to a quartile, by assigning 1.0, 0.75, 0.5, and 0.25 contingent on whether its value is more than the third quartile, median, first quartile, or none of the three, respectively, for that given property. Overall, $\textbf{X}$ has dimension $n \times 6$.

#### Labels

To determine labels for the PA criteria, we find the median of the number of link removals necessary for the size of the largest connected component to become half its original value. Since a vulnerable network is likely to fragment easily, a network with a lower than the median number of links removed gets a label 0, and 1 otherwise, in the ($n \times 1$) vector, $\textbf{y}$.

Finally, for each PA criterion, we calculate the coefficients from the discriminant analysis (see Fig. 2). The coefficients reflect the role of each topological property on network vulnerability for PA-based link removal criteria.

**Table 1:** Properties of the networks. *N*: number of nodes, *E*: number of edges, *T*: transitivity, *r*: assortativity coefficient, *M*: modularity, *D*: density, *ASP*: average shortest path, *d*: diameter. B.N: Biological Network, Br.N: Brain Network, G.N: Grid Network, S.N: Social Networks, Sy.N: Synthetic Network, L.N: Linguistic Network, T.N: Trade Network.

Network	Type	|*N*|	|*E*|	*ASP*	*r*	*D*	*d*	*M*	*T*
Dolphins^37^	B.N.	62	159	3.357	-0.044	0.084	8	0.495	0.309
*E. coli*^38^	B.N.	1477	3658	3.579	-0.351	0.003	9	0.584	0
bn-macaque-rhesus_brain_2^39,40^	Br.N.	91	582	1.868	-0.77	0.142	3	0.079	0
bn-cat-mixed-species_brain_1^39–41^	Br.N.	65	730	1.7	-0.025	0.351	3	0.295	0
Circuits s838_st^42^	G.N.	512	1324	1.994	-0.24	0.01	3	0.802	0.573
Circuits s420_st^42^	G.N.	252	644	1.987	-0.243	0.02	3	0.746	0.565
Circuits s208_st^42^	G.N.	122	189	4.928	-0.002	0.026	11	0.679	0.058
Arenas email	S.N.	1133	5451	3.606	0.078	0.009	8	0.582	0
fb-pages-food^39^	S.N.	620	2102	5.089	-0.028	0.011	17	0	0
Facebook0^43^	S.N.	324	2514	3.753	0.233	0.048	11	0.445	0.426
Facebook107^43^	S.N.	1034	26749	2.952	0.431	0.05	9	0.458	0.504
Facebook348^43^	S.N.	224	3192	2.523	0.223	0.128	9	0.248	0.49
Facebook686^43^	S.N.	168	1656	2.425	0.084	0.118	6	0.29	0.454
Facebook414^43^	S.N.	148	1692	2.692	0.304	0.156	7	0.544	0.646
Facebook1684^43^	S.N.	786	14024	3.042	0.33	0.045	10	0.521	0.746
Soc-firm-hi-tech^39^	S.N.	33	123	1.769	-0.256	0.233	2	0.313	0.372
Karate^44^	S.N.	34	78	2.408	-0.476	0.139	5	0.381	0.256
Soc-tribes^39^	S.N.	17	76	1.449	-0.079	0.559	2	0.169	0.527
Barabasi_albert_graph^2^	Sy.N.	500	1491	3.267	-0.096	0.012	5	0.39	0.028
Word adjacencies^45^	L.N.	112	425	2.536	-0.129	0.068	5	0.293	0.157
Polbooks	T.N.	105	441	3.079	-0.128	0.081	7	0.569	0

## Results

We discuss the network datasets used in the study, followed by the experimental findings from their vulnerability analysis. Table. 1 summarizes the topological indicators of the networks; Table 2 provides a ranked list of the indicators either in an increasing or decreasing order to aid the explanation of their roles on overall network vulnerability to attack models.

**Complex network datasets.** Several different types of complex networks have been used for this study. The network types used herein are social networks, biological networks (such as genetic regulatory networks, ecological networks, and brain networks), synthetic networks (e.g., Barabasi-Albert networks), and collaboration networks (such as co-authorship networks). The differences between the different network types reflect variant connectivity patterns exhibited within each network.

1. *Dolphins*^37^: This is a network that shows the frequent interaction among 62 bottlenose dolphins.
2. *Escherichia coli GRN*^38^: This is a biological network that represents the interactions among genes and transcription factors of *E. coli* to regulate the organism’s functionality. Nodes represent genes and transcription factors, while edges represent their interactions.
3. *bn-macaque-rhesus_brain_2*^39,40^: This network depicts the neural connections, or connectome, present in the brain of rhesus macaque monkeys.
4. *bn-cat-mixed-species_brain_1*^39–41^: represents the connectome (neural connection network) of cortical areas from the brain of cats.
5. *Circuits (s208_st, s420_st, and s838_st)*^42^: This represents electrical circuits networks, obtained from (http://www.weizmann.ac.il/mcb/UriAlon/download/collection-complex-networks).
6. *Arenas email*: The network represents the email communication system of the University Rovira i Virgili, located in Tarragona, southern Catalonia, Spain. In this network, each user is represented as a node, and an edge between two nodes indicates that at least one email was sent between them; data can be obtained from (http://deim.urv.cat/~aarenas/data/welcome.htm).
7. *fb-pages-food*^39^: This network represents the interactions among Facebook pages of multiple food companies that were collected in the year 2017.
8. *Facebook (0,107, 348, 414, 686, and 1684)*^43^: These networks are extracted from Facebook and represent the social interactions among its users, where nodes represent friends and edges represent various forms of social interactions such as liking, sharing, or messaging.
9. *Soc-firm-hi-tech*^39^: This network depicts the relationships of friendship among the employees of a small high-tech computer firm.
10. *Karate*^44^: This is a network of 34 members belonging to a Karate club, with each member being classified into a group based on their affiliation status. The grouping emerged from a dispute between the club’s instructors and administrators. Wayne W. Zachary collected and analyzed this dataset during the period from 1970 to 1972.
11. *Soc-tribes*^39^: This network illustrates the cultural and linguistic groups present in the central Highlands of New Guinea, showcasing their varying degrees of similarity and difference.
12. *Barabasi-Albert*^2^: The preferential attachment algorithm is utilized to generate random scale-free networks. The algorithm is based on the concept that the likelihood of a new node establishing a connection with an existing node is proportional to the number of connections that the existing node has.
13. *Word Adjacency*^45^: This network depicts the adjacency of noun-noun, adjective-noun, or adjective-adjective words in the novel “David Copperfield”. The nodes in the network correspond to nouns and adjectives, while the edges represent their adjacency.
14. *Polbook*: This network (obtained from the website http://www.orgnet.com/) comprises US politics books, where nodes indicate the books, and edges indicate the frequent co-purchasing of books on amazon.com by the same buyer.

**Table 2:** Ranking the most influential topological indicators of the networks: assortativity coefficient *r* (in decreasing order); and modularity *M* and transitivity *T* (in increasing order).

Network	*r* (High to low)	*M* (Low to high)	*T* (Low to high)
Facebook107	1	12	16
Facebook1684	2	14	21
Facebook414	3	15	20
Facebook0	4	11	13
Facebook348	5	4	15
Facebook686	6	5	14
Arenas email	7	17	5
Circuits s208_st	8	19	8
bn-cat-mixed-species_brain_1	9	7	3
fb-pages-food	10	1	1
Dolphins	11	13	11
Soc-tribes	12	3	17
Barabasi_albert_graph	13	10	7
Polbooks	14	16	4
Word adjacencies	15	6	9
Circuits s838_st	16	21	19
Circuits s420_st	17	20	18
Soc-firm-hi-tech	18	8	12
E. coli	19	18	6
Karate	20	9	10
bn-macaque-rhesus_brain_2	21	2	2

**Figure 3:**
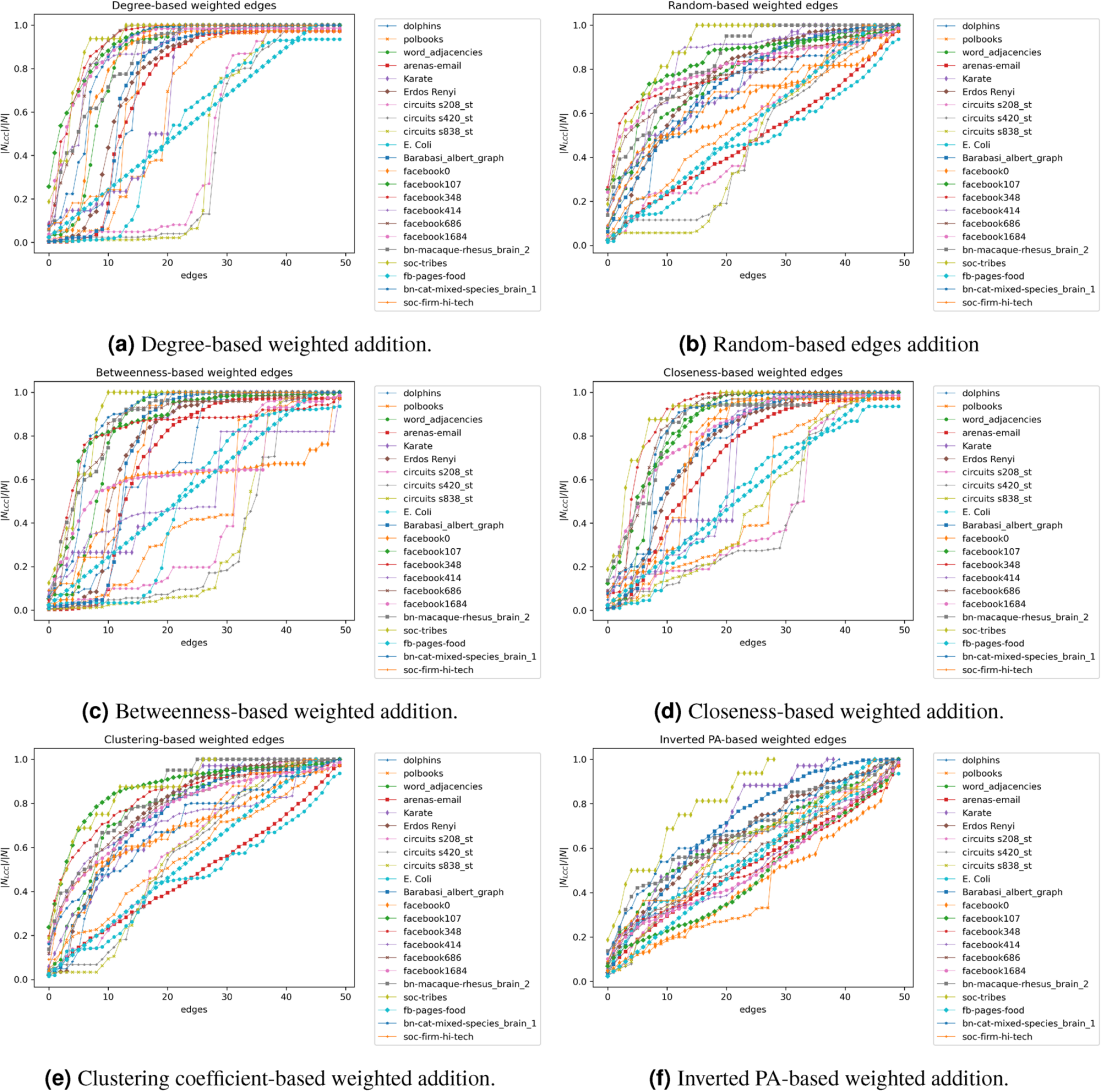
Measuring the percolation of the network using different growing models. Edges are weighted and added based on RND, PA, BPA, CPA, CcPA, and iPA models.

### Performance of the networks on edge additions

We examine how the choice of topological indicators affects their network robustness. For a network, the links are ranked by their degree (DNA), betweenness (BNA), closeness (CNA), clustering coefficient (CcNA), and inverse preferential attachment (iPA) scores (refer to section Network growth and depletion models on attack models). Starting with an empty network, we add links in batches in decreasing order of scores for that attack model till all the links are restored. The robustness of a network is measured by how quickly it reaches the highest size of its largest connected component, i.e., a vulnerable network is likely to remain fragmented for the longest batches of link restoration. We delve into the topological indicators of networks with the highest and least robustness.

For random link restoration (RND), DNA, and BNA, we find *Soc-tribe*, followed by *bn-cat-mixed-species_brain_1*, *bn-macaque-rhesus_brain2* and *Facebook686*, is the most robust. We report in Table 2 that these networks are characterized by low modularity (*M*) rank ($\le 7$). On the other hand, *Circuits s838_st* and *Circuits s420_st*, both characterized by a combination of high *M* and low assortativity *r* rank, are the most vulnerable (see Figs. 3a,b,c).

**Figure 4:**
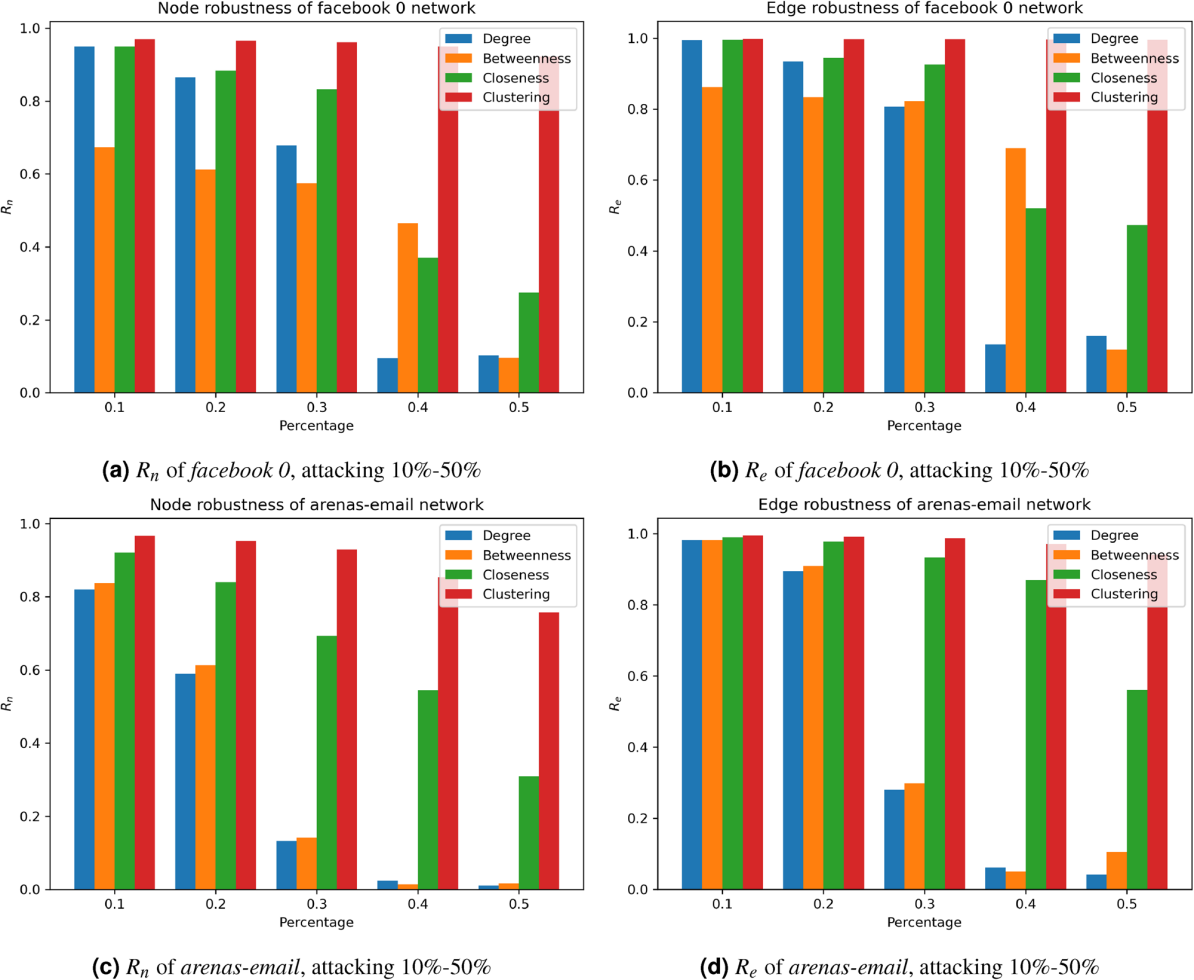
Node and edge robustness ($R_n, R_e$) of two sample networks (facebook 0 and arenas-email). Each network is attacked by removing (10%, 20%, 30%, 40%, and 50%) of the nodes, using high (1- degree, 2- betweenness, 3- closeness, and 4- clustering coefficient) values.

Coming to the other attack models, namely, CNA, CcNA, and iPA, low *M* is associated with high robustness: *Soc-tribe* with modularity rank 3 again is the most robust; *Facebook348*, *bn-macaque-rhesus_brain2*, and *Karate club* are the next best (modularity $\le 9$). Conversely, *E. coli*, *Circuits s420_st*, *Circuits s208_st*, *Facebook107*, *Arenas-email*, and *Poolbook*, which have high *M* as a common network property, are the most vulnerable (Fig. 3d,e,f). Overall, this suggests that network modularity is a key indicator of robustness during network growth. This is because modular networks are intrinsically clustered and specific links need to be added to restore connectivity. To strengthen this observation, we report low *relative* modularity and clustering to be negatively correlated with node and edge robustness metrics (refer to Eqs. (7, 8) of main text) in Supplementary 1. We extend this experiment, by considering the effect of the same indicators during the inverse process where links are removed from networks. Figure 1 in Supplementary 2 illustrates that the results are similar yet not identical: assortativity plays an even more pivotal role than modularity in the robustness during depletion.

### Choice of attack model

We study the effect of centrality-based attack models on the robustness of the networks, each network was attacked using each one of the given attack models (DNA, BNA, CNA, and CcNA). In each attack model (*m*), we removed five different percentages (10%, 20%, 30%, 40%, and 50%) of the nodes that resulted in an attacked network of ($G^m$) and measured the performance of the node and edge robustness metrics of the attacked networks (refer to Eqs. (7 and 8)). Figure 4. depicts node and edge robustness of the different attack models on two selected networks (Facebook 0 and arenas-email networks), where betweenness- and degree-based attacks impact network robustness the most, while clustering-based node attacks have the most negligible effect.

### Quantifying the effect of indicators on network vulnerability

We employ partial least squares discriminant analysis (PLS-DA) to measure the effect of the network indicator features, namely, clustering coefficient (or transitivity), modularity, density, assortativity, efficiency, diameter, and average shortest path (ASP) on its vulnerability to myriad preferential attachment (PA)-based link failure models. Recall from our discussion in section Partial least squares discriminant analysis, the labels used in the PLS-DA analysis are the minimum number of links that need to be removed for the size of the largest connected component of a network to become half its original value. Since a robust network will need commensurately more removals to disintegrate, the PLS-DA coefficients ultimately measure the contribution of the topological indicators on robustness. Specifically, a high (positive) coefficient alludes to a higher contribution of an indicator toward robustness. Our analysis shows that *low relative assortativity* and *high relative modularity* are hallmarks of vulnerable networks, across all failure models. This is because, highly modular, disassortative networks form dense clusters, each containing local hub nodes connected to low-degree nodes. The removal of links connecting such clusters creates fragments of disconnected clusters^46^.

**Table 3:** Partial least squares discriminant analysis for the effect of the topological indicators of a network on its vulnerability to the following attack or failure scenarios: degree (DPA), betweenness (BPA), closeness (CPA), clustering coefficient (CcPA), random (RND) and inverted preferential attachment (iPA).

Topological indicators	Failure models					
	DPA	BPA	CPA	CcPA	RND	iPA
Transitivity	−0.231	−0.014	−0.176	−0.025	0.041	−0.014
Modularity	−0.172	−0.201	−0.222	−0.095	−0.105	−0.201
Density	−0.121	−0.070	−0.071	0.118	0.030	−0.070
Assortativity	0.100	0.315	0.059	0.254	0.182	0.315
Efficiency	−0.093	−0.070	−0.163	−0.144	−0.013	−0.070
Diameter	−0.088	−0.049	−0.038	−0.020	−0.018	−0.049
ASP	0.031	−0.006	−0.007	−0.034	−0.024	−0.006

As pointed out in section Introduction, the contributions of the indicators toward network robustness are relative. For example, a high coefficient of assortativity suggests that a network that is neither assortative nor disassortative (i.e., $r \approx 0$) is more robust than a disassortative network ($r \approx -1$) for that given attack scenario. Table 3 shows that for both degree and betweenness PA-based link removals, *relatively* low modularity, high assortativity, low-density, and low transitive networks exhibit the least vulnerability. For closeness PA-based link removal, low modularity, coupled with low modularity and efficiency, lends the highest robustness. On the other hand, the removal of links with a high clustering coefficient can be guarded against by the presence of high density and assortativity with low modularity. Finally, for random and inverse PA-based link removal, less modular and *relatively* assortative networks exhibit the least vulnerability. Analysis in Supplementary 4 shows networks with the least modularity retain the low diameter and remain unfragmented for the highest proportion of random node removals.

## Application of the study

We discuss the following three key takeaways from this vulnerability analysis, before presenting an illustrative example of how the results from the proposed study can be harnessed to achieve robust networking solutions.

1. *Analysis of network vulnerability and attack models.* Our research has highlighted that network vulnerability is related to the nature of the attack model. This insight underscores the importance of tailoring network defense strategies to specific threats. While high assortativity and low modularity have shown effectiveness in enhancing robustness, the size of the largest connected component can also be influenced by topological factors, including degree, betweenness, closeness, and cluster-based link removals, acknowledging that attackers possess a range of strategies to disrupt network functionality.
2. *Applications in critical infrastructure and beyond.* The multifaceted perspective on attack strategies highlights the need for network defenders to anticipate and prepare for a wide array of potential threats. Specifically, the implications of our findings extend to a variety of critical domains, where network connectivity is paramount and any disruption can have severe consequences. For example, (a) in the context of a power grid, this could involve rapid repairs or rerouting of electricity flows to minimize downtime during an attack; (b) in telecommunication networks, it may entail redundant communication channels or protocols to ensure uninterrupted service. In disaster response and environmental monitoring, maintaining network connectivity is vital for timely data collection and emergency coordination; and (c) for military and defense networks, ensuring connectivity is crucial for effective communication, surveillance, and command and control.
3. *Partial least squares discriminant analysis (PLS-DA) as an effective tool to build network resilience strategies.* This work introduces the application of Partial Least Squares Discriminant Analysis (PLS-DA) as a tool for identifying links that need to be restored to preserve connectivity. This analytical approach can be invaluable for network operators and security experts in critical sectors. By using PLS-DA, they can pinpoint potential links that are critical to network integrity.

**Figure 5:**
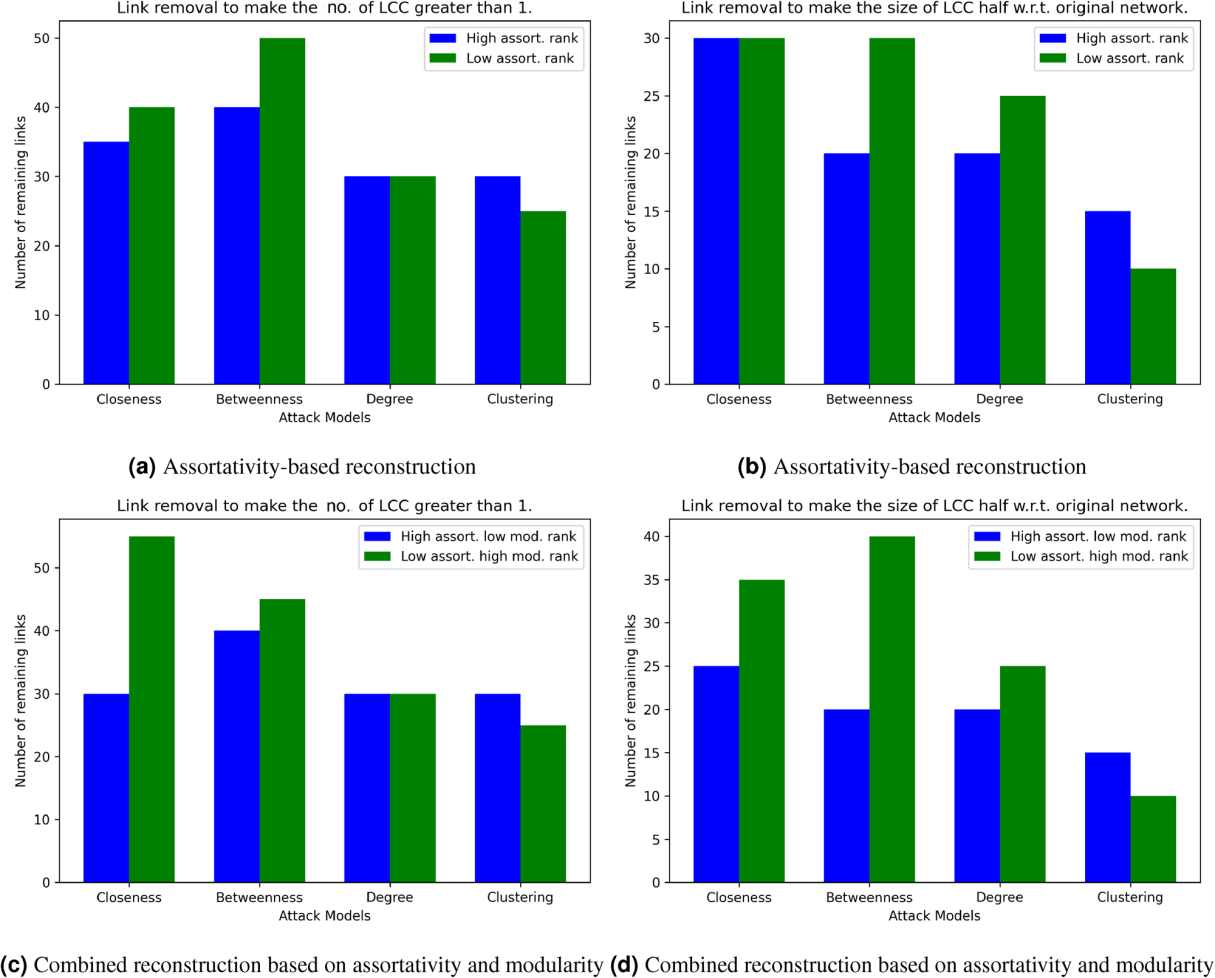
Example of network attack and reconstruction on a complete 30-node network: assortativity-based reconstruction after (**a**) link removal to make the number of largest connected component (LCC) exceed 1 (**b**) link removal to make the size of LCC half with respect to original networks; combined reconstruction based on assortativity and modularity (**c**) link removal to make the number of LCC exceed 1 and (**d**) link removal to make the size of LCC half with respect to original networks.

In light of the aforementioned applications, let us discuss an *illustrative example* of how the topological indicators can be leveraged in network reconstruction. We take a complete toy network of 30 nodes and apply closeness, betweenness-, degree- and clustering-based attacks, i.e., CPA, BPA, DPA, and CcPA, respectively, to remove 10 links and restore 5 links based on two scenarios: assortativity and combination of assortativity and modularity, as follows.

1. *Assortativity.* Recall that the assortativity of most networks studied in this work ranges from low assortativity $r \approx 0$ to disassortative $r < 0$ (refer to Table 1 for details). Given a depleted network *G*(*V*, *E*), we separately add the link *e* that (a) maximizes the score $\frac{1}{|r_{G(V, E \cup e)}|}$ to encourage the resultant network to have $r \approx 0$ and (b) minimizes $r_{G(V, E \cup e)}$ to cause the resultant network to be disassortative.
2. *Combination of assortativity and modularity.* Again, as per Table 1, the modularity *M* ranges between $\approx 0$ to positive. As a preprocessing step, all unconnected node pairs (i.e., potential links) are scored by the assortativity and modularity upon their addition to their depleted networks. Both of these scores are scaled using min-max normalization before separately adding the link *e* that maximizes (a) $\frac{r_{G(V, E \cup e)}}{M_{G(V, E \cup e)}}$ to encourage the resultant network to have high assortativity and low modularity and (b) $\frac{M_{G(V, E \cup e)}}{r_{G(V, E \cup e)}}$ to make the network low in assortativity yet high in modularity.

For both networks in scenarios 1 and 2, we record the number of links remaining when (a) the network begins to fragment, i.e., the number of the largest connected component (LCC) becomes greater than 1 and (b) the size of LCC becomes half of that of the original complete network. Notably, a robust network should take more link removals to disintegrate, suggesting that fewer links should remain in the depleted networks. Figure 5a,b show that the networks regenerated based on high assortativity rank criteria have fewer remaining links under most attacks, corroborating the PLS-DA findings that low assortativity rank is related to network vulnerability. For the same reason, the networks regenerated based on combined high assortativity and modularity criteria have fewer remaining links (both when LCC exceeds 1 and LCC becomes half the original size) under most attacks (Fig. 5c,d). Thus, the network operator can use high assortativity ranks or combine high assortativity and low modularity ranks to determine the links to be restored in order to enhance network connectivity in the face of specific attacks.

## Discussion

We explored the effect of topological indicators on the vulnerability of complex networks to diverse node and link attack models, such as degree-based attack (DNA), betweenness-based node attack (BNA), closeness-based node attack (CNA), clustering coefficient-based node attack (CcNA), and random node attack (RNA). We carried out extensive experiments with growth- and depletion-based attack models and partial least squares-discriminant analysis (PLS-DA) to pinpoint indicators that influence network vulnerability more than others. Almost all the analyses reveal high network modularity and low assortativity as indicators of network vulnerability (or low robustness). These observations are consistent with existing literature, where assortative networks have been shown to exhibit greater robustness against targeted attacks^46,47^. The vulnerability analysis, in conjunction with node and link robustness metrics, suggests that networks with high assortativity rank or low network modularity (see Table. 2), or both, such as *bn-cat-mixed-species_brain_1* or *Facebook (107, 686)* are less likely to be fragmented than those with low assortativity and/or high modularity, like *Escherichia coli GRN* and *Circuits s838_st*.

These findings open up new directions to achieve network robustness based on the nature of the attack models being applied. Through the use of regression and discriminant analysis methods (PLS-DA), we elucidate that it is possible to quantify the individual effect of topological indicators on robustness in an attack-specific fashion. This facilitates the design of adaptive optimization and machine learning (ML) models to grow networks that are robust or avoid network fragmentation in the face of targeted attacks. The growth- and depletion-based analysis discussed in section Performance of the networks on edge additions and Supplementary 2 underpin the fact that growth and depletion are not identical with respect to the influential indicators. For instance, high modularity drives network vulnerability during growth, while assortativity is more critical during depletion. Finally, it is worth noting that this line of research will be particularly effective when the nature of the attack is not well-defined. An attacker may calculate a combined score of multiple centrality-based scores to determine the critical components or resort to a mixed strategy to weaken network recovery efforts. Such attacks could be offset by the use of computational models that distributively and periodically learn the regression coefficients for topological indicators contributing to robustness in large complex networks.

### Supplementary Information

Supplementary Information.

## Data Availability

The datasets used, generated, and/or analyzed during the current study are available in the GitHub repository https://github.com/almusawiaf/vulnerability.
