# Supplementary material for: Examining indicators of complex network vulnerability across diverse attack scenarios
Source: Sci Rep. 2023 Oct 24;13:18208. doi: 10.1038/s41598-023-45218-9 (PMC10598276; doi:10.1038/s41598-023-45218-9)
Supplement: Supplementary file 1 — Supplementary Information. [file 41598_2023_45218_MOESM1_ESM.pdf]

# Supplementary Information

**Ahmad F. Al Musawi, Satyaki Roy, and Preetam Ghosh**

## 1 Correlation analysis of networks' features and robustness

In order to gain insights into the association between the various characteristics of the network and the robustness of nodes and edges (represented as $R_n$ and $R_e$, respectively), a correlation analysis was performed on the properties of networks discussed in Sec. 3.1 of the main text. The Pearson correlation coefficients among the different network features were also presented in Table 1, where a positive correlation indicates a strong association between the variables with both tending to increase or decrease together; conversely, a negative correlation signifies that as one variable increases, the other decreases. A correlation value approaching zero indicates the absence of any discernible relationship between the variables.

We report that both node and edge robustness are correlated with the same set of features. $R_n$ and $R_e$ show a moderate level of correlation with the number of nodes, edges, average shortest path, density, and diameter. We find that robustness is negatively correlated with low modularity and clustering, but it is positively correlated with assortativity, hinting that less modular, assortative networks may exhibit better robustness to node and edge failure. The robustness of the network is also connected to the size of the network with a long diameter, which could be a function of low modularity and assortativity.

| | $\|V\|$ | $\|E\|$ | $ASP$ | $D$ | $r$ | $d$ | $ACC$ | $T$ | $M$ |
|---|---|---|---|---|---|---|---|---|---|
| $R_n$ | 0.94 | 0.89 | 0.43 | -0.58 | 0.099 | 0.41 | -0.38 | -0.45 | -0.29 |
| $R_e$ | 0.76 | 0.97 | 0.29 | -0.42 | 0.36 | 0.41 | -0.1 | -0.052 | -0.33 |

**Table 1.** Pearson correlation between the features of the networks and network's node and edge robustness, $R_n$,$R_e$ respectively. $N$: number of nodes, $E$: number of edges, $ACC$: average clustering coefficient, $r$: assortativity coefficient, $M$: modularity, $D$: density, $ASP$: average shortest path, $d$: diameter.

## 2 Robustness of networks under edge attacks

The performance evaluation of networks involves a deliberate focus on edge removal. This evaluation quantifies the impact of removing edges on the size of the largest connected component relative to the overall size of the network (i.e., $\frac{|V_{LCC(G)}|}{|V_G|}$ where $V_{LCC(G)} \subset V_G$). To assign weights to the edges, various node centralities, such as degree, betweenness, closeness, and clustering coefficient centralities, are employed. Initially, the centrality of nodes is computed using one of these measures. Each edge is then assigned a weight equal to the product of the centrality values of the nodes it connects, following Eq. 12. Subsequently, the edges are systematically removed in decreasing order of weight. To facilitate presentation, the network's edges are divided into 50 batches, and the performance of the network is evaluated after the removal of each individual batch. However, to introduce randomness into the simulation, the sequence of edges is shuffled before the random-based attack model is executed.

Fig. 1 shows how networks respond to link removal and Table 2 in the main text shows the ordered list of networks based on three influential predictors, namely, modularity (ranked high to low); assortativity, and clustering coefficient (ranked low to high). The most (and least) robust networks preserve the size of the largest connected component for the longest (and shortest) batches of link removals. For random (RND), degree-based (DNA), and betweenness (BNA) link failures (Figs. 1a, 1b and 1c), we find the *bn_cat_mixed-species_brain_1*, and *Facebook107* exhibit the best robustness, followed by *Facebook686* and Barabasi networks; most of these networks have high assortativity ranks. On the other hand, *Soc-tribe*, *Karate club*, and *Circuits s838_st* have low assortativity ranks ($\geq 12$) and are the worst-performing, for most of RND, DNA, and BNA.

Looking at *closeness* (CNA) and *clustering coefficient* (CcNA)-based failures (see Figs. 1d and 1e), assortativity and modularity once again emerge as key indicators of vulnerability. The network *bn-macaque-rhesus_brain_2* is the most robust, followed by *Facebook686* and *bn_cat_mixed-species_brain_1*. These networks possess a low modularity rank ($\leq 7$). Conversely, *Soc-tribe* and *karate club*, marked by a low assortativity rank, are the worst performing. Finally, for *inverse preferential attachment*, *Facebook686*, *Facebook107* are most robust, while *bn-macaque-rhesus_brain_2* and *Circuits s838_st* are the worst. The best- and worst-performing networks show high and low assortativity ranks, respectively (Fig. 1f).

## 3 Robustness of networks under node attacks

We conducted an additional experiment to assess network connectivity measured by the size of the largest connected component (LCC) normalized by the number of nodes $N$ when subjected to node removal. The nodes for removal are chosen based on degree, betweenness, closeness, and clustering coefficients. For clarity, we divided the centrality-based sorted list of network nodes into 50 batches and evaluated the network's performance after removing each batch individually. We intuit that the most

robust networks are likely to preserve their LCC for the longest batches of node removal. Fig. 2 illustrates that the *Facebook networks (414, 1684, 348)* demonstrated the highest robustness, followed by *bn-cat-mixed-species_brain_1*. Most of these networks exhibit high assortativity rank or low modularity ranks within the network datasets summarized in Table 2 of the main text. Conversely, the *fb-pages-food* network displayed the lowest level of robustness in all cases; *E. coli* underperformed particularly in the DNA and BNA models, and *Soc-tribes* and *bn-macaque-rhesus_brain_2* exhibited poor performance in the CNA and CcNA models, respectively. These networks share low assortativity and high modularity characteristics.

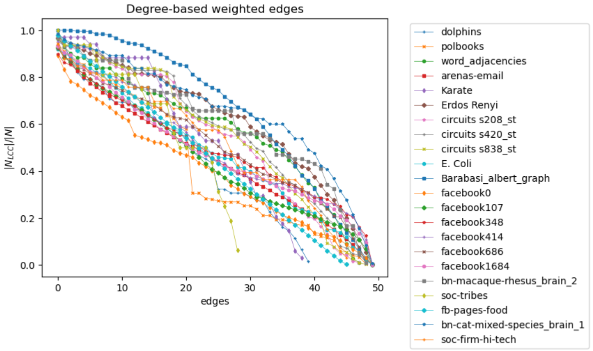


**(a)** Degree-based weighted removal.

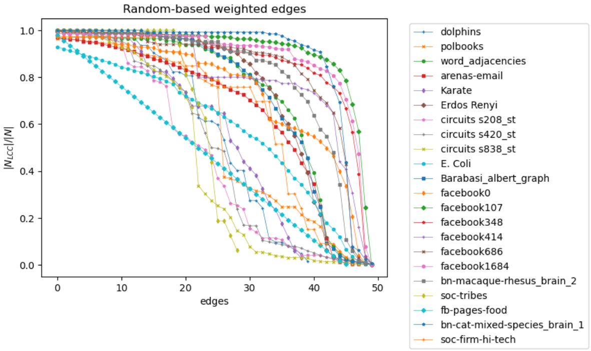


**(b)** Random-based edges removal.

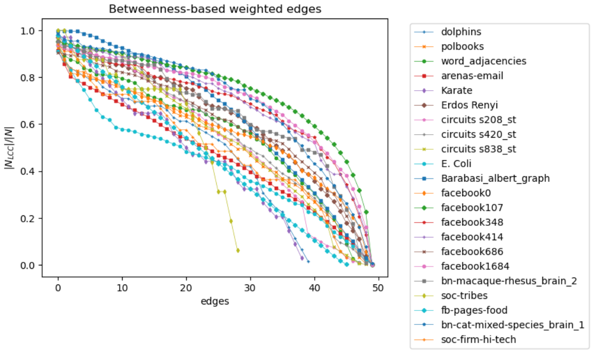


**(c)** Betweenness-based weighted removal.

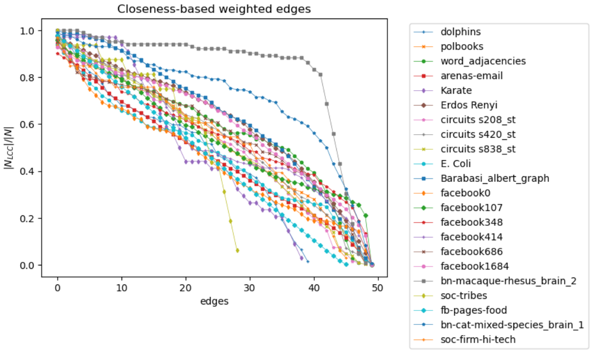


**(d)** Closeness-based weighted removal.

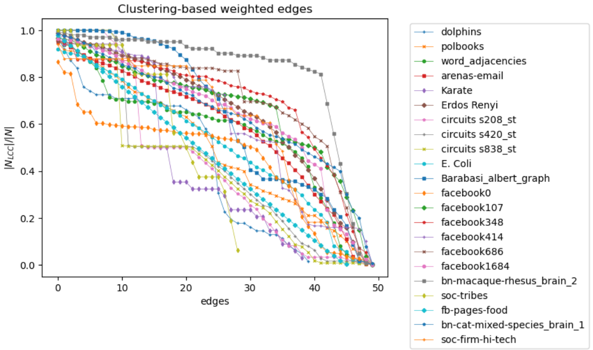


**(e)** Clustering coefficient-based weighted removal.

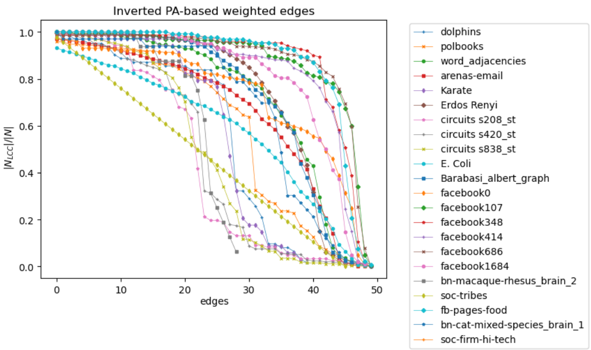


**(f)** Inverted preferential attachment-based weighted removal.

**Figure 1.** Analysis of network robustness (in terms of the size of the largest connected component normalized by the number of nodes $N$) using different attack models. Edges are weighted and removed based on RND, PA, BPA, CPA, CcPA, and iPA.

## 4 Effect of Random Node Removal on Diameter

We study the effect of random node removal on diameter. Fig. 3a shows that the evolving diameter of the largest connected component stays stable upon node removal. We also record the effect of node removal on diameter. Since the diameter of the network is considered undefined when the network ceases to be one connected component, we end the diameter curve when the network fragments into more than one component. Fig. 3b shows that *fb-pages-food* has the lowest modularity (see Table 2 in

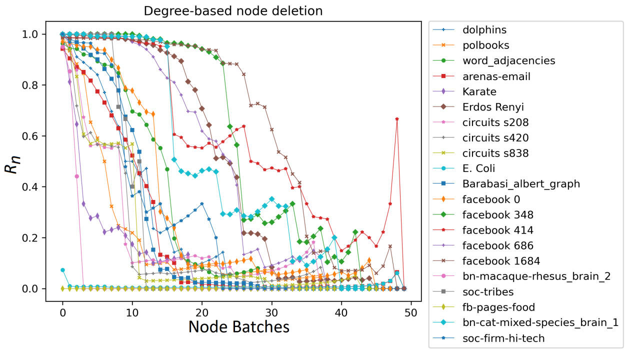


**(a)** $R_n$, Degree-based nodes removal.

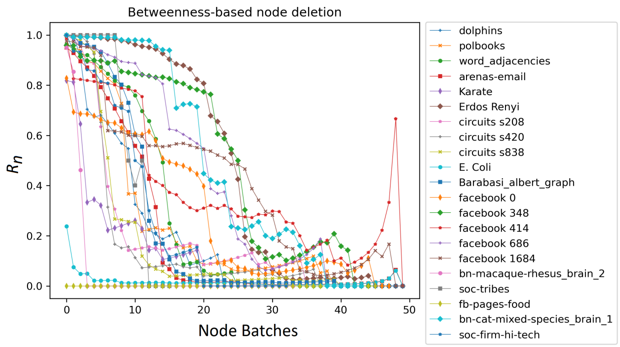


**(b)** $R_n$, Betweenness-based nodes removal.

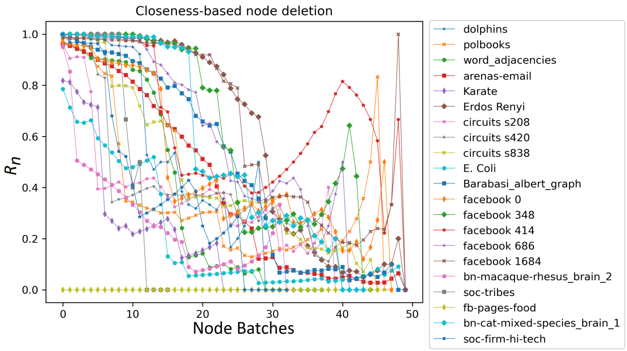


**(c)** $R_n$, Closeness-based nodes removal.

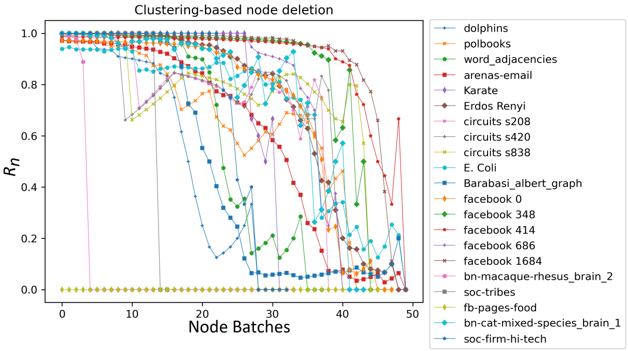


**(d)** $R_n$, Clustering coefficient-based nodes removal.

**Figure 2.** Analysis of network robustness (in terms of the size of the largest connected component normalized by the number of nodes $N$) using different attack models. Nodes are removed based on their degree, betweenness, closeness, and clustering coefficient centralities.

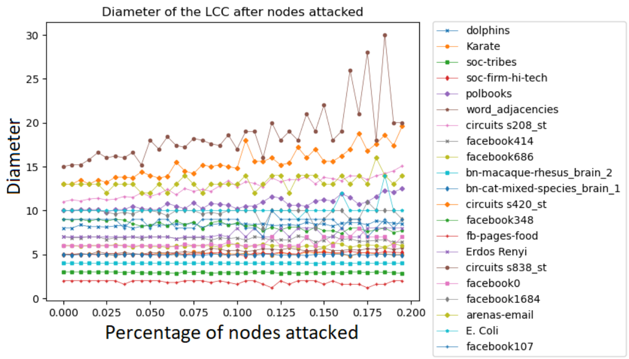


**(a)** Diameter of LCC

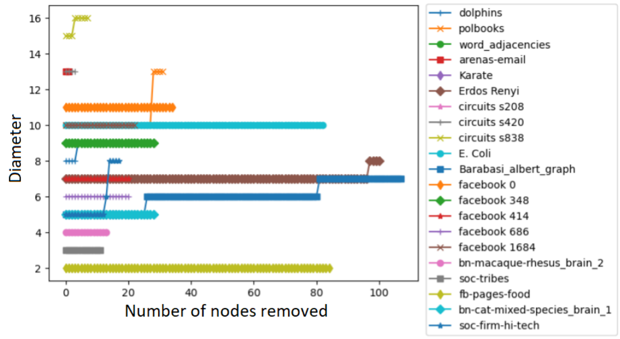


**(b)** Diameter of entire network

**Figure 3.** Measuring the diameter of the network after the random removal of nodes using a specific percentage ($X$-axis). We measured the average diameter of 25 different experiments for each percentage.

the main text) and maintains the lowest diameter for the longest time, whereas *Arenas email* and *Circuits s838_st* have the highest diameter and fragment the earliest. Both these networks have the worst-ranking modularity, i.e., 17 and 21, respectively.
